# Vertigo and dizziness due to vertebrobasilar TIA: a prospective study

**DOI:** 10.3389/fstro.2024.1429068

**Published:** 2024-10-02

**Authors:** Arlindo C. Lima Neto, Ji-Soo Kim, Wanderley Marques Bernardo, Roseli Saraiva Moreira Bittar

**Affiliations:** ^1^Department of Otorhinolaryngology, University of São Paulo, São Paulo, Brazil; ^2^Department of Neurology, Seoul National University College of Medicine, Seoul National University Bundang Hospital, Seoul, Republic of Korea; ^3^Medical School, University of São Paulo, São Paulo, Brazil

**Keywords:** TIA, vascular, vertigo, dizziness, treatment, vertebrobasilar, cohort

## Abstract

**Purpose:**

Prospective studies on vascular vertigo and dizziness (VVD) due to vertebrobasilar transient ischemic attack (VBTIA) have been sparse. This study aimed to characterize clinical features, response to treatments, and prognostic factors of VVD due to VBTIA using a cohort established in 2021.

**Methods:**

We recruited 103 patients (58 female individuals, 56.3%), with a mean age of 70.9 ± 9.3 years (range = 37–85), between January 2021 and January 2024. All patients met the diagnostic criteria of “Probable transient VVD” published by the Bárány Society. The mean interval from symptom onset to recruitment was 11.8 months (range = 0.5–72). Treatments followed the current American Heart Association–American Stroke Association's Guidelines for Prevention of Stroke in Patients with Stroke and Transient Ischemic Attack. Patients with recurrent strokes among TIAs, and patients who were already taking an antithrombotic agent and should maintain the same regimen were excluded.

**Results:**

Imbalance (46.7%) and vertigo (39.8%) were the most frequent symptoms. The duration of attacks was <1 min in 35 patients (33.9%), 1–10 min in 34 patients (33.0%), 10–60 min in 15 patients (14.6%), and >60 min in 19 patients (18.5%). Trigger factors were reported in 20 patients (19.4%), which included eccentric neck position in 12 patients (11.7%), physical exercise in four patients (3.9%), positional changes in three patients (2.9%), and eccentric neck position and physical exercise in the remaining patient (0.9%). The frequency of attacks before the medication was 1 or <1/month in 32 (31.0%) patients, 1–4/month in 44 (42.7%) patients, 4–8/month in 21 patients (20.4%), and daily in six patients (5.9%). The treatment regimens were aspirin in 57 patients (55.3%), clopidogrel in 19 patients (18.5%), aspirin plus clopidogrel in 25 patients (24.3%), and rivaroxaban in two patients (1.9%). The attacks were reduced by 93.2% [IC 95% (88.34, 98.06), number needed to treat: 1] during the median follow-up of 12 months (range = 2–36 months). Only seven (6.8%) patients experienced a new attack with the medication. No prognostic factors could be identified for the recurrences.

**Conclusion:**

VVD due to VBTIA has a broad clinical spectrum. Secondary stroke prevention is effective in VVD due to VBTIA even though no prognostic factors could be identified for symptom recurrence.

## Introduction

Two important research lines have been structured for vascular vertigo and dizziness (VVD). One is related to vertigo or dizziness due to stroke or transient ischemic attack (TIA) with the primary focus on their diagnosis in the emergency department (ED). The other line is dedicated to the prognosis of vertigo and dizziness due to vertebrobasilar TIAs (VBTIAs).

Diagnosing TIA is challenging because it is mostly based on the patient's symptoms, the physician's ability to interpret them correctly, and the exclusion of other diagnoses (Tuna and Rothwell, 2021; Yao and Zu, 2023). Many studies still refer to the National Institute of Neurological Disorders and Stroke (NINDS) criteria for diagnosing TIA (Paul et al., 2013). However, those do not properly embrace vertigo and dizziness as symptoms of TIA. Thus, the studies on vertigo and dizziness due to VBTIA have been sparse (Markus et al., 2013; Pollak, 2012; Fife et al., 1994; Jenkins et al., 2001).

In 2021, our group established a cohort of patients with VVD and treated them according to the Guidelines for Prevention of Stroke in Patients with Stroke and Transient Ischemic Attack published by the American Heart Association and American Stroke Association (AHA/ASA) (Kleindorfer et al., 2021). The majority of patients suffered from recurrent VBTIAs. In 2022, the diagnostic criteria for VVD were launched by the Bárány Society, which summarized and facilitated the characterization of this clinical entity and injected fresh air into our cohort (Kim et al., 2022).

This study aimed to characterize the clinical features, response to treatment, and prognostic factors of VVD due to VBTIA that could interfere with the outcomes after treatments.

## Methods

This cohort study started in January 2021 at the Neuro-otology Service of the Clinics Hospital of the Medical School of São Paulo University. In this article, we present the data collected until January 2024. All patients were first evaluated in an ED or an outpatient clinic.

### Patient selection

The inclusion criteria were

- Episodic vertigo, dizziness, or unsteadiness lasting <24 h.
- At least one of the following: (a) focal central neurological symptoms or severe postural instability during the attack; (b) new-onset moderate to severe craniocervical pain during the attack, (c) increased risk for vascular events, for example, ABCD2 score of 4 or greater or atrial fibrillation; (d) significant (>50%) narrowing of an artery in the vertebrobasilar (VB) system; and (e) isolated spontaneous episodic vertigo in older adults with no other possible causes.
- Another disease or disorder that better accounted for the symptoms.
- Patient eligibility for antiplatelet or anticoagulant therapy according to the 2021 AHA/ASA Guidelines for the Prevention of Stroke in Patients with Stroke and Transient Ischemic Attack.

The exclusion criteria were

- Patients who present with findings of acute ischemia on the cranial magnetic resonance imaging (MRI), including diffusion-weighted MRI (DWI-MRI) at recruitment.
- Patients who also fulfilled the diagnostic criteria for other neurotological diseases that may present acute or recurrent transient dizziness/vertigo (benign paroxysmal positional vertigo, Ménière's disease, vestibular migraine, vestibular paroxysmia, vestibular neuritis, persistent postural-perceptual dizziness, postural hypotension, presbyvestibulopathy, motion sickness, and cervical dizziness), neurodegenerative disorders, or cognitive deficits.
- Patients already taking antiplatelets or anticoagulants who should maintain the same medication.
- Patients whose first attack was a vertebrobasilar stroke, taking into account the findings of previous ischemia on the brain DWI-MRI.
- Patients who had suffered from multiple strokes (two or more), regardless of the region involved, taking into account the findings of previous ischemia on the brain DWI-MRI.
- Patients who had other symptoms during the follow-up that suggested another etiology of dizziness or vertigo.
- Patients who did not complete the entire evaluation.
- Patients who did not sign the informed consent form.

**Figure 1:**
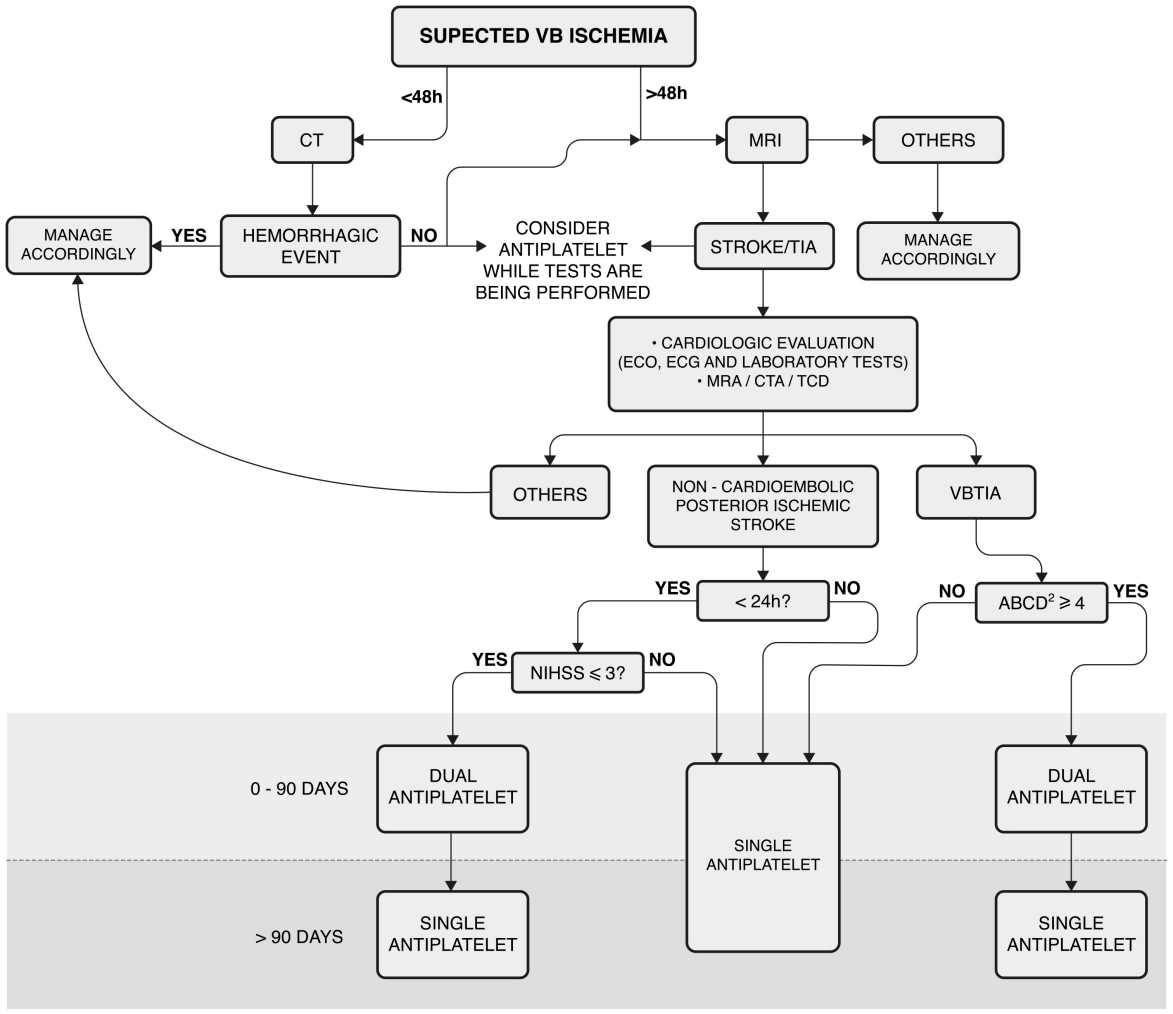
Algorithm for managing patients with vascular vertigo/dizziness. This algorithm was not applicable to patients receiving acute thrombolysis. Laboratory tests included complete blood count, troponin, prothrombin time, partial thromboplastin time, glucose, hemoglobin A 1c, creatinine, and fasting and non-fasting lipid profiles. MRI: cranial diffusion-weighted magnetic resonance; VB, vertebrobasilar; CT, cranial computed tomography; ECO, echocardiography; ECG, electrocardiogram. MRA, cranial and cervical magnetic resonance angiography; CTA, cranial and cervical computed tomography; TCD, transcranial Doppler ultrasonography; VBTIA, vertebrobasilar transient ischemic attack; Dual antiplatelet, aspirin plus clopidogrel; NIHSS, National Institutes of Health Stroke Scale.

To confirm the diagnosis of VVD and to exclude other possible causes, all participants underwent a complete otolaryngological and neurological examination. The evaluation included anamnesis, ocular misalignment, spontaneous, gaze-evoked, positional nystagmus, bedside head impulse test (HIT), video-oculography (VOG), tonal and vocal audiometry, and tympanometry. In some cases, vestibular-evoked myogenic potentials (VEMPs) and video HIT (vHIT) were also performed. All patients also had a cardiovascular evaluation for ABCD2, which included ambulatory blood pressure monitoring, Holter electrocardiography, and Doppler echocardiography. Tilt testing was also performed when autonomic dysfunction was suspected. All participants were subjected to DWI-MRI and magnetic resonance angiography (MRA) or computed tomographic angiography (CTA) of the VB territory. Figure 1 shows the algorithm adopted to manage the patients in this cohort.

### Stroke in this data set

To emphasize how we handled the report or radiological evidence of stroke, we highlight the following:

- If a patient reported a stroke before the onset of vertigo/dizziness and no imaging of previous ischemia was found on brain DWI-MRI, this was considered a previous comorbidity.
- If there was imaging evidence of a previous single (non-acute) ischemic focus in the supratentorial region, this was also considered a previous comorbidity.
- If the recruitment DWI-MRI revealed one focus of previous (non-acute) VB ischemia in a patient with recurrent vertigo/dizziness attacks (three or more), we classified this as a posterior circulation stroke among the episodes of TIA. However, if the patient reported two attacks of vertigo/dizziness and presented findings of a previous VB stroke, we concluded that the first attack was a stroke; therefore, this patient was excluded.
- If the recruitment DWI-MRI revealed multiple ischemic foci, regardless of the region or the history of symptoms, the patient was excluded.
- If the recruitment DWI-MRI revealed an acute ischemia or hemorrhage, the patient was also excluded.

### Therapeutic intervention

The first-line treatment for the patients with VVD was aspirin (100 mg/day) or clopidogrel (75 mg/day), depending on allergy, bleeding, preference, or any other reported side effects. Dual antiplatelet therapy (DAPT) was used for a maximum of 90 days, as shown in Figure 1. If patients were already taking an antiplatelet drug (aspirin or clopidogrel) and had a clinical indication for DAPT, aspirin plus clopidogrel was prescribed. If the most appropriate treatment was an anticoagulant, it was used as a monotherapy. Vascular risk factors, such as lifestyle, hypertension, blood lipid and glucose levels, obesity, and obstructive sleep apnea, were also monitored (Kleindorfer et al., 2021).

After starting the medication, patients were monitored monthly for the first 6 months and every 3 months afterward. If a patient experienced a new attack during the follow-up, the algorithm was restarted (Figure 1).

### Variables

We also gathered data regarding age, sex, interval from the first symptom onset, features of dizziness/vertigo (imbalance, spinning, unsteadiness, and positional vertigo), duration, triggers, other hypoflow symptoms, frequency of episodes (before and after the medication), vascular comorbidities, follow-up duration, interval from the treatment initiation and a new attack, and imaging findings. For didactic purposes, the findings of MRA and CTA in the VB system were grouped under “VB large artery disease.”

For a new VVD attack (stroke or VBTIA) during the medication period, the following prognostic factors were considered: age, sex, duration and frequency of attacks before treatments, comorbidities (highlighting arterial hypertension), VB large artery disease, the interval from the first symptom onset, and treatment regimens.

### Ethical considerations

All patients voluntarily agreed to participate in the study and signed an informed consent form. The local ethics committee approved this study, and our protocol adhered to the tenets of the Declaration of Helsinki.

### Statistical analyses

The data collected (variables) were described qualitatively, primarily to demonstrate a risk reduction in dizziness or vertigo after treatment. There was no sample estimation because the sample consisted of all patients treated in a given period. Therefore, the cohort represented a varied period of follow-up. A survival curve was developed to consider censured data from patients who presented with recurrent stroke or TIA, who were lost the follow-up, who reached the last day of follow-up in January 2024, and who were followed by less than the median of the follow-up period.

The main analysis was univariate and related to the presence or absence of the following prognostic factors during follow-up and after treatments: age, sex, duration and frequency of attacks, comorbidities, VB large artery disease, and treatment regimens. The risk was calculated using a 95% confidence interval and the Mantel–Haenszel test. The benefit of treatment was expressed as the number needed to treat (NNT). The statistical power of “VB large artery disease” was also calculated.

Some variables were dichotomized for the prognostic analysis. The means were used to consider the presence of these variables (higher or shorter, less or more, or younger or older). The means were used for age (70.9 years), duration of attacks (18.2 min), frequency of attacks (4.2 episodes per month), and interval from the onset (11.8 months).

To calculate the hazard ratio for recurrences during the follow-up, the group with events in all at-risk patients and the group without events in all at-risk patients were compared (Georgiev, 2024).

## Results

**Figure 2:**
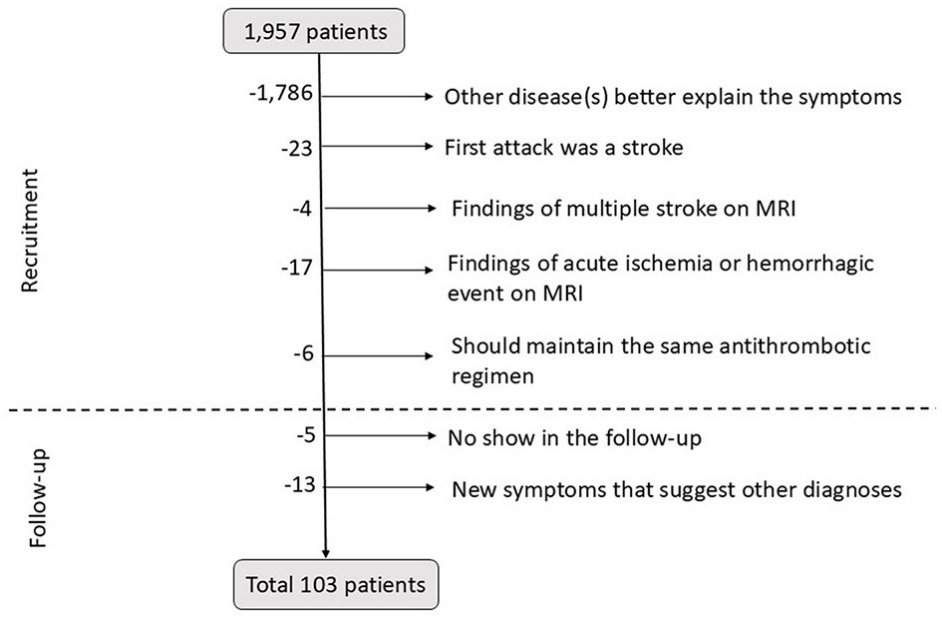
Diagram of patient's exclusion at the recruitment and during the follow-up period. MRI, magnetic resonance imaging.

Out of 1,957 patients, 103 were finally included in the study. Among these patients, 58 (56.3%) were female individuals, with a mean age of 70.9 ± 9.3 years (ranging from 37 to 85 years). In all, 69 patients (67.0%) were recruited from the outpatient clinic, and 34 patients (33.0%) were recruited from the ED. All data are summarized in Supplementary Table 1. Figure 2 shows the selection process.

### Symptoms and comorbidities

The mean interval from the symptom onset to recruitment was 11.8 months, ranging from 0.5 to 72. The vestibular symptoms included dizziness/imbalance in 48 patients (46.7%), spinning vertigo in 41 patients (39.8%), unsteadiness in 11 patients (10.6%), and positional vertigo in three patients (2.9%). The duration of attack was < 1 min in 35 patients (33.9%), 1–10 min in 34 patients (33.0%), 10–60 min in 15 patients (14.6%), and >60 min in 19 patients (18.5%). Triggering factors were reported in 20 patients (19.4%), which included eccentric neck position (such as head rotation, flexion, or extension) in 12 patients (11.7%), physical exercise (fast walking, climbing stairs, and carrying bags) in four patients (3.9%), positional changes in three patients (2.9%), and both eccentric neck position and physical exercise in the remaining patient (0.9%). The frequency of attacks before the medication was 1 or < 1/month in 32 patients (31.0%), 1–4/month in 44 patients (42.7%), 4–8/month in 21 patients (20.4%), and daily in six patients (5.9%).

The most frequently associated symptoms were transient visual loss (*n* = 31, 30%), syncope (*n* = 28, 27.2%), diplopia (*n* = 11, 10.7%), balance-related fall (*n* = 10, 9.7%), dysphagia (*n* = 6, 5.8%), and new-onset cranial pain (*n* = 5, 4.8%). Furthermore, 16 patients reported no symptoms (15.5%), and 22 had two or more (21.3%).

Table 1 presents the prevalence of comorbidities. We also highlight that 10 patients (9.7%) had no vascular risk factors, 33 (32.0%) had 2, and 39 (37.8%) had 3 or more.

**Table 1:** Prevalence of comorbidities in the patients (*n* = 103).

**Comorbidities**	***N***	**%**
Arterial hypertension	74	71.8
Dyslipidemia	47	45.6
Diabetes	46	44.6
Ischemic heart disease	11	10.6
Cardiac arrhythmia	11	10.6
Previous anterior circulation stroke	10	9.7
Smoking	4	3.8
Kidney insufficiency	3	2.9

Thirty-three patients presented two comorbidities, 39 suffered three or more, and 10 did not report any.

### Imaging findings

A total of 18 patients (17.4%) exhibited evidence of a previous VB stroke on their MRIs, and none reported an attack lasting more than 24 h. The ischemia observed on MRIs involved the cerebellum in 10 patients (9.7%), the thalamus in three patients (2.9%), the pons in three patients (2.9%), the medulla and pons in 1 patient (0.9%), and the midbrain in the remaining patient (0.9%).

Abnormalities of VB arteries (“VB large artery disease”) were identified on MRA or CTA in 39 patients (37.8%). The most common abnormalities included distal vertebral artery stenosis in 12 patients (11.6%), severe vertebral artery hypoplasia in 10 patients (9.7%), proximal vertebral artery stenosis in eight patients (7.8%), bilateral vertebral artery hypoplasia associated with fetal-type variants of the posterior cerebral artery in three patients (2.9%), and vertebral artery dissection in two patients (1.9%).

### Treatment regimens and follow-up duration

The treatment regimens included aspirin in 57 patients (55.3%), clopidogrel in 19 patients (18.5%), DAPT in 25 patients (24.3%), and rivaroxaban in two patients (1.9%). Additionally, 19 patients (18.5%) were already using monotherapy and had their treatment adjusted to include DAPT.

The median follow-up duration was 12 months, ranging from 2 to 36 months. For 43 patients (41.7%), the follow-up period was longer than 12 months.

### Outcomes

**Figure 3:**
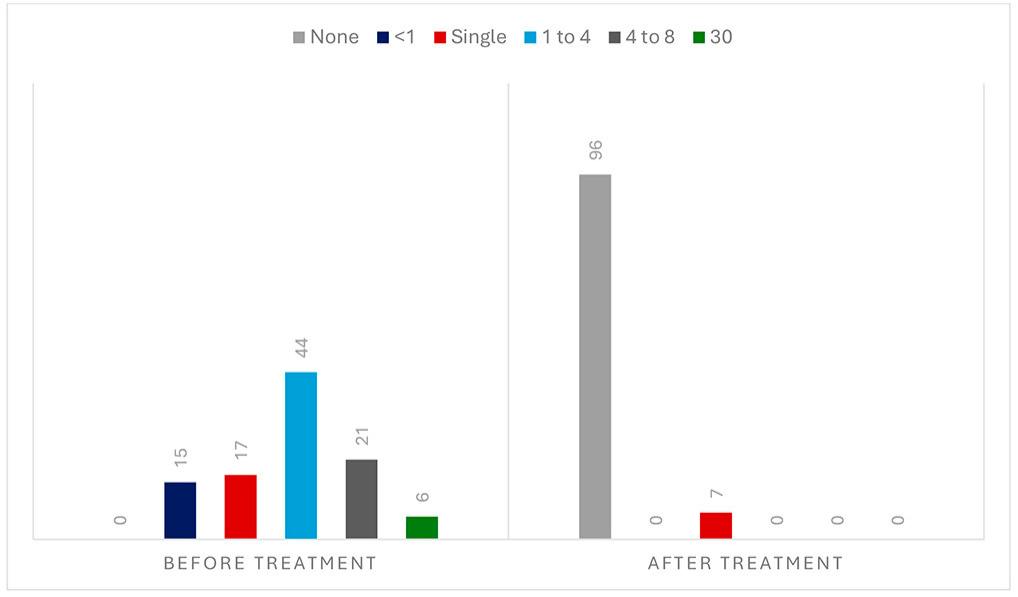
Frequency of attacks before and during the medication (*n* = 103).

**Figure 4:**
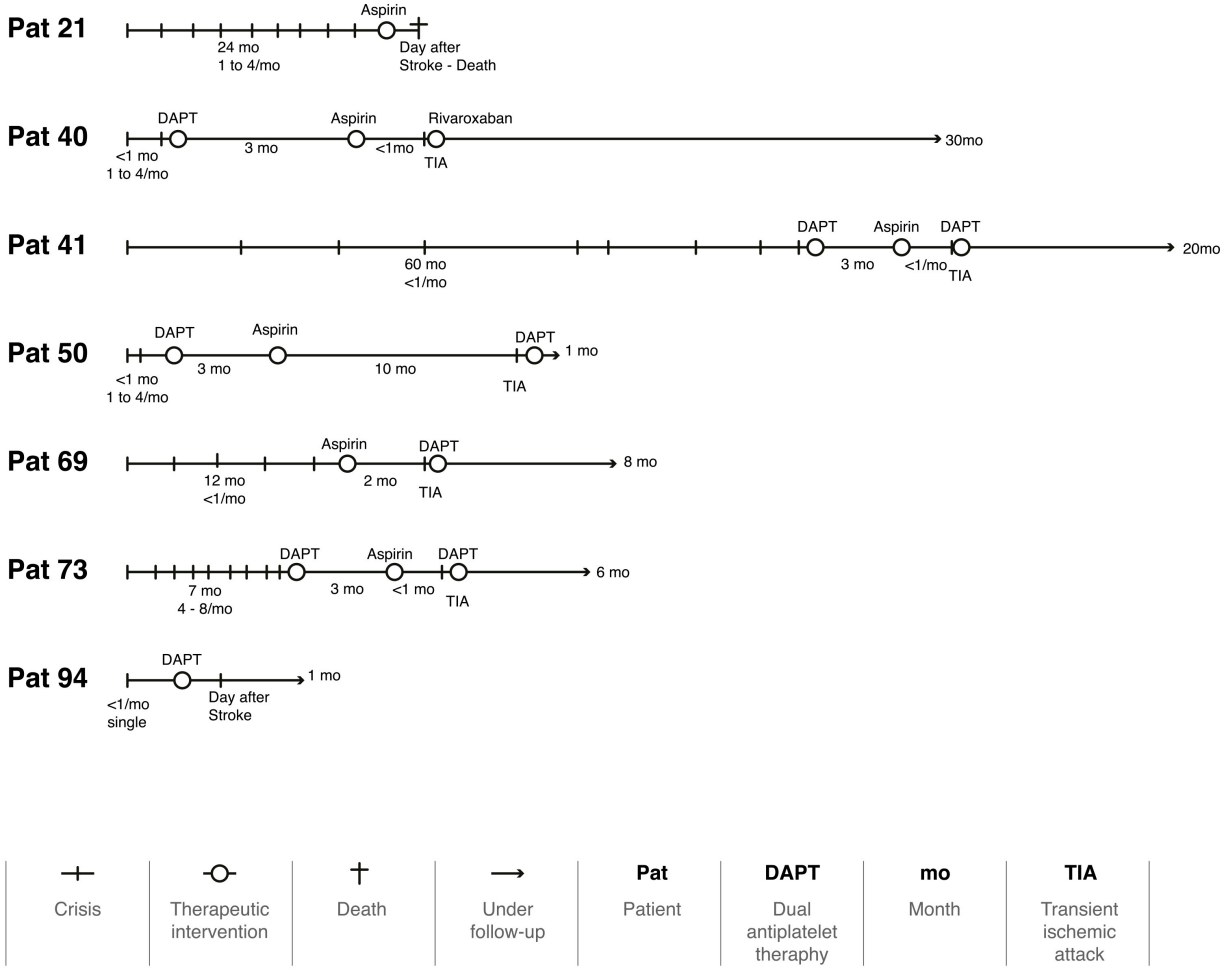
The timeline of seven patients with a recurrence during the follow-up period.

During follow-up, 96 patients (93.2%) had no further attacks [IC 95% (88.34, 98.06), NNT: 1], but seven (6.8%) had a single recurrence (Figure 3). Two had a stroke the day after the medication, and one died of complications. Four had a TIA, three of them when the regimen was switched from DAPT to monotherapy. Figure 4 illustrates the evolution of these seven patients.

**Figure 5:**
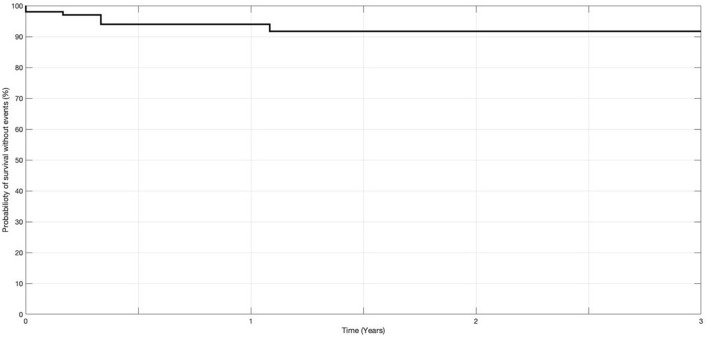
Probability of recurrences of vertebrobasilar TIA at 36 months of medication (*n* = 103).

Figure 5 represents the Kaplan–Meier survival curve for recurrences of VBTIA during the follow-up period. All but one recurrent attack occurred during the first year of follow-up. The probability of VBTIA recurrence decreased during the 36 months of treatments.

Almost all prognostic factors analyzed (sex, age, duration and frequency of attacks, presence and number of comorbidities, hypertension, and interval from the onset) were not significantly associated with the outcomes, except for the presence of VB large artery disease, which demonstrated a difference of 37.5% [CI 95% (27.8%, 47.1%)] favoring the absence of events, with three patients with a large artery disease requiring treatment to avoid an event (Table 2). The statistical power for this result was 51.9%.

**Table 2:** Prognostic factors in vertebrobasilar TIA (*n* = 103).

**Prognostic factor analyzed**	**Statistical test**		
	**Risk ratio**	**Risk difference (%)**	***p*** **-value**
Sex (female vs. male)	1.0 (0.2, 4.4)	0.2 (−9.5, 10.0)	0.48
Age group (younger vs. older)	2.3 (0.5, 11.2)	5.2 (−4.3, 14.7)	0.15
Attack duration (short vs. high)	1.2 (0.2, 6.0)	1.4 (−8.6, 11.4)	0.40
Attack frequency (less vs. more)	2.1 (0.3, 16.9)	4.2 (−5.2, 13.5)	0.23
Comorbidities (≥3 vs. ≤ 2)	2.5 (0.5, 9.3)	6.6 (−4.8, 18.0)	0.10
Comorbidities (≥2 vs. ≤ 1)	2.8 (0.3, 22.5)	5.5 (−3.2, 14.3)	0.15
Arterial hypertension (presence vs. absence)	1.1 (0.2, 5.2)	0.5 (−10.6, 11.6)	0.47
Time from onset (short vs. high)	1.2 (0.3, 5.0)	1.1% (−6.0, 11.3)	0.41
VB large artery disease (yes vs. no)	0.0 (0.0, ?)	−37.5 (−27.8, −47.1)	0.04

The number of comorbidities (≥3, ≤ 2, ≥2, < 1) are as listed in Table 1. Means of dichotomized variables: age (70.9 years), attack duration (18.2 min), attack frequency (4.2 episodes per month), and time from first symptom onset (11.8 months).

VB, vertebrobasilar.

Although not all patients reached 36 months of follow-up, the hazard ratio was 1.4%, demonstrating that 98.6% of patients would have a reduction in events within the maximum follow-up period.

## Discussion

Up to 25% of strokes are preceded by a TIA (Giles and Rothwell, 2007; Halmagyi, 2017). The early risk of stroke after a TIA is high, requiring urgent investigation and treatment (Johnston et al., 2000; Rothwell et al., 2007; Lavallée et al., 2007). In addition, considering the evidence of high stroke risk in transient VVD, active secondary prevention measures, including antithrombotic agents, statin, and risk factor control, deserve special attention (Yao and Zu, 2023).

In this sample, we observed a balanced distribution of sex, age, and prevalence of comorbidities, similar to the distribution found in VVD studies conducted in ED settings (Filippopulos et al., 2022; Kuroda et al., 2017) and studies including both VB and anterior circulation strokes (Andersen et al., 2010).

Other authors have presented the elapsed time from symptom onset to the diagnostic evaluation and treatment (Tuna and Rothwell, 2021; Pollak, 2012; Fife et al., 1994). The mean interval from symptom onset to recruitment was 11.8 months. However, four patients presented with symptoms that lasted longer than 5 years (Supplementary Table 1). Many factors may favor the infratentorial region for TIA rather than stroke. One is the high proportion of the white matter and the vascular distribution in the cerebellum and brainstem because collaterals can be formed by the rich web from the main trunks (Supplementary Figure 2) (Mattle et al., 2011). This anatomical background probably explains why TIAs and isolated transient neurological attacks (TNAs) are more common in the 90 days preceding a vertebrobasilar stroke compared to their occurrence before a carotid stroke [noted in 59 out of 275 patients before a vertebrobasilar stroke vs. 65 out of 759 patients before a carotid stroke; odds ratio (OR) 2.92, 95% CI (1.99, 4.28), *p* < 0.0001] (Paul et al., 2013). This same rationale supports a longer time window for the treatment of VB strokes, such as those caused by occlusion of the basilar artery (Mattle et al., 2011; Merwick and Werring, 2014; Räty et al., 2024; Alemseged et al., 2023). Another possible protective factor found in the participants in our study was the prior use of antiplatelet therapy, even with a regimen insufficient to prevent VVD attacks (Tuna and Rothwell, 2021). However, both physicians and patients probably neglected this condition, which contributed to the long interval between the onset of symptoms and the first evaluation in patients with recurrent VBTIA. It is estimated that only 22% of patients with TNA from the brainstem sought medical care before stroke. In only one case (2%), a vascular cause was suspected by their physician (Paul et al., 2013).

Vertigo/dizziness was the only symptom in 16 of our patients. Some studies have reported that isolated episodic vertigo was the only manifestation in 21%−62% of patients with a presumptive diagnosis of VBTIA and that 29% of patients with VB infarction had a history of isolated episodic vertigo (Kim et al., 2022; Gomez et al., 1996; Grad and Baloh, 1989). Even in VB strokes, obvious focal neurological signs were absent in more than half of the individuals presenting with acute vestibular syndrome (Tarnutzer et al., 2011).

Another diagnostic challenge was that nearly 10% of patients had no comorbidities other than age. Conducting a thorough search for other associated symptoms of hypoflow, undiagnosed risk factors, and other possible etiologies, such as postural hypotension and medication side effects, among others, is imperative before considering prophylactic treatments.

Findings on MRA or CTA scans may also contribute to the suspicion of VBTIA. In one study, patients with TNA were more likely to show arterial stenosis ≥50% in the posterior circulation than those with “classic” TIA [according to the NINDS; 84 of 467 (18%) vs. 80 of 896 (9%); odds ratio (OR) 2.21, 95% CI (1.59, 3.08), *p* < 0.0001] (Tuna and Rothwell, 2021). The correlation between the luminal irregularities in the posterior circulation and VVD is well established (Choi et al., 2017; Lima Neto et al., 2017). In our sample, 39 (37.8%) patients presented with vertebrobasilar artery abnormalities, with vertebral artery stenosis and severe hypoplasia being the most frequent. There is substantial evidence for vertebral artery hypoplasia as a risk factor for posterior circulation ischemia, especially when other vascular risk factors coexist (Kleindorfer et al., 2021; Katsanos et al., 2013; Park et al., 2007). Nevertheless, in patients with a high suspicion of VBTIA and without findings in MRA or CTA (64 patients in our sample, 62.2%), abnormalities of the secondary arteries and focal atherosclerosis should be considered in the pathogenesis of symptoms (Park et al., 2014; Kim et al., 2011; Lima Neto et al., 2020).

Even though we achieved an acceptable average follow-up time, a longer follow-up period would be beneficial for monitoring vascular and chronic disorders. Throughout this period, the use of aspirin, clopidogrel, DAPT, and anticoagulants appear to be an effective approach in treating VVD patients, as only seven patients (6.8%) had a new attack. Considering that two of these cases had their new attack the day after the prescription, it is likely that they were not yet under the effect of aspirin (Figure 4). Another three patients experienced a new attack as soon as they were switched from DAPT to a single therapy. Using anticoagulants and stent placement in patients with VVD who have not responded to antiplatelet therapy has been reported in some case series; however, there is no established guidance on how to implement this approach (Fife et al., 1994; Jenkins et al., 2001). Our results suggest that this management of the treatment strategies must be individualized for each patient. This will certainly be the focus of future studies.

However, a reasonable criticism would be that we excluded patients whose first crisis was a stroke, patients with recurrent strokes, and patients who were already on an antithrombotic regimen that did not need to be changed according to the AHA/ASA guidelines. These excluded cases may have had a worse prognosis with more recurrences. We decided not to include them because we focused our method on VBTIA and on evaluating the possible reduction in the number of VVD attacks with treatment. For example, we would not know whether the dizziness was related to recurrent ischemic events or sequelae of stroke. Similarly, the response of patients whose regimens were neither initiated nor changed by us could not be evaluated. As this cohort study is ongoing, we aim to have a larger sample of these individuals whose treatment regimen remained unchanged to form a comparison group.

This sample did not allow us to identify factors that could increase the risk of developing a new attack in patients receiving treatments. Even in other prospective study with 17,009 years of follow-up, the number of cases of vertigo/dizziness (considered “non-consensual TIA”) was too small to reliably estimate the risks (Tuna and Rothwell, 2021). Only one variable, “VB large artery disease,” showed significance (*p* = 0.04) but favored the absence of events. Even though the mechanism is unknown, we hypothesize that this protective effect against a new attack may have been due to neovascularization facilitated by the chronic hypoperfusion in these patients because most of them presented vertebral artery hypoplasia. However, given the low statistical power of this result, a confounding factor must be considered due to the small number of patients with a recurrence. Our data will be reviewed again when we have a larger sample size and longer follow-up.

We emphasize that this cohort study was conducted in a Latin American country with a predominantly mixed ethnicity and a tropical climate. Future comparisons must consider these characteristics.

Some implications of our findings for clinical practice are as follows: (a) we demonstrated that secondary prophylaxis is the correct treatment for vertigo/dizziness attacks due to VBTIA; (b) we also confirmed that “non-consensual VBTIA” is “VBTIA indeed” in terms of treatment efficacy in preventing stroke, aligning with authors who previously used this term (Tuna and Rothwell, 2021); (c) patients with VVD due to VBTIA should not be routinely excluded from clinical trials and other research studies; (d) medical education is needed to provide physicians with the knowledge and tools to identify and treat these patients; and (e) public education is also essential to encourage people to seek medical attention after an episode of dizziness or vertigo.

## Conclusion

VBTIA has a broad clinical spectrum. In this study, the predominant symptom was weekly imbalance attacks lasting up to 10 min accompanied by transient visual loss, syncope, diplopia, and balance-related falls.

Adhering to AHA/ASA guidelines, secondary prevention treatments using aspirin, clopidogrel, or DAPT were quite effective, with 93.2% of patients showing no recurrences during the follow-up period. However, this sample size was insufficient to identify factors that might increase the risk of a new attack in treated patients.

## Data Availability

The original contributions presented in the study are included in the article/Supplementary material, further inquiries can be directed to the corresponding author.

## References

[B1] Alemseged F. Nguyen T. N. Coutts S. B. Cordonnier C. Schonewille W. J. Campbell B. C.. (2023). Endovascular thrombectomy for basilar artery occlusion: translating research findings into clinical practice. Lancet Neurol. 22, 330–337. 10.1016/S1474-4422(22)00483-5 36780915

[B2] Andersen K. K. Andersen Z. J. Olsen T. S. (2010). Age- and gender-specific prevalence of cardiovascular risk factors in 40,102 patients with first-ever ischemic stroke: a nationwide Danish study. Stroke 41, 2768–2774. 10.1161/STROKEAHA.110.595785 20966413

[B3] Choi J. H. Park M. G. Choi S. Y. Park K. P. Baik S. K. Kim J. S.. (2017). Acute transient vestibular syndrome: prevalence of stroke and efficacy of bedside evaluation, Stroke 48, 556–562. 10.1161/STROKEAHA.116.015507 28100765

[B4] Fife T. D. Baloh R. W. Duckwiler G. R. (1994). Isolated dizziness in vertebrobasilar insufficiency: clinical features, angiography, and follow-up. J. Stroke Cerebrovasc. Dis. 4, 4–12. 10.1016/S1052-3057(10)80139-9 26487528

[B5] Filippopulos F. M. Strobl R. Belanovic B. Dunker K. Grill E. Brandt T.. (2022). Validation of a comprehensive diagnostic algorithm for patients with acute vertigo and dizziness. Eur. J. Neurol. 10, 3092–3101. 10.1111/ene.15448 35708513

[B6] Georgiev G. Z. (2024). Hazard Ratio Calculator. Available at: https://www.gigacalculator.com/calculators/hazard-ratio-calculator.php (accessed September 15, 2024).

[B7] Giles M. F. Rothwell P. M. (2007). Risk of stroke early after transient ischaemic attack: a systematic review and meta-analysis. Lancet Neurol. 6, 1063–1072. 10.1016/S1474-4422(07)70274-0 17993293

[B8] Gomez C. R. Cruz-Flores S. Malkoff M. D. Sauer C. M. Burch C. M. (1996). Isolated vertigo as a manifestation of vertebrobasilar ischemia, Neurology 47, 94–97. 10.1212/WNL.47.1.94 8710132

[B9] Grad A. A. Baloh R. W. (1989). Vertigo of vascular origin. Clinical and electronystagmographic features in 84 cases. Arch. Neurol. 46, 281–284. 10.1001/archneur.1989.00520390047014 2919982

[B10] Halmagyi G. M. (2017). Brainstem stroke preceded by transient isolated vertigo attacks. J. Neurol. 264, 2170–2172. 10.1007/s00415-017-8610-0 28894926 PMC5617861

[B11] Jenkins J. S. White C. J. Ramee S. R. Collins T. J. Chilakamarri V. K. McKinley K. L.. (2001). Vertebral artery stenting. Catheter. Cardiovasc. Interv. 54, 1–5. 10.1002/ccd.1228 11553939

[B12] Johnston S. C. Gress D. R. Browner W. S. Sidney S. (2000). Short-term prognosis after emergency department diagnosis of TIA. JAMA 284, 2901–2906. 10.1001/jama.284.22.2901 11147987

[B13] Katsanos A. H. Kosmidou M. Kyritsis A. P. Giannopoulos S. (2013). Is vertebral artery hypoplasia a predisposing factor for posterior circulation cerebral ischemic events? A comprehensive review. Eur. Neurol. 70, 78–83. 10.1159/000351786 23816871

[B14] Kim D. U. Han M. K. Kim J. S. (2011). Isolated recurrent vertigo from stenotic posterior inferior cerebellar artery. *Otol*. Neurotol. 32, 180–182. 10.1097/MAO.0b013e3181f6ca2f 20856154

[B15] Kim J. S. Newman-Toker D. E. Kerber K. A. Jahn K. Bertholon P. Waterson J.. (2022). Vascular vertigo and dizziness: diagnostic criteria. J. Vestib. Res. 32, 205–222. 10.3233/VES-210169 35367974 PMC9249306

[B16] Kleindorfer D. O. Towfighi A. Chaturvedi S. Cockroft K. M. Gutierrez J. Lombardi-Hill D.. (2021). 2021 Guideline for the prevention of stroke in patients with stroke and transient ischemic attack: a guideline from the American Heart Association/American Stroke Association. Stroke 52, e364–e467. 10.1161/STR.0000000000000375 34024117

[B17] Kuroda R. Nakada T. Ojima T. Serizawa M. Imai N. Yagi N.. (2017). The TriAGe+ score for vertigo or dizziness: a diagnostic model for stroke in the emergency department. J. Stroke Cerebrovasc. Dis. 26, 1144–1153. 10.1016/j.jstrokecerebrovasdis.2017.01.009 28256416

[B18] Lavallée P. C. Meseguer E. Abboud H. Cabrejo L. Olivot J. M. Simon O.. (2007). A transient ischaemic attack clinic with round-the-clock access (SOS-TIA): feasibility and effects. Lancet Neurol. 6, 953–960. 10.1016/S1474-4422(07)70248-X 17928270

[B19] Lima Neto A. C. Bittar R. S. M. Gattas G. S. Bor-Seng-Shu E. Oliveira M. L. Monsanto R. C.. (2017). Pathophysiology and diagnosis of vertebrobasilar insufficiency: a review of the literature. Int. Arch. Otorhinolaryngol. 21, 302–307. 10.1055/s-0036-1593448 28680502 PMC5495592

[B20] Lima Neto A. C. Bor-Seng-Shu E. Oliveira M. L. Macedo-Soares A. Topciu F. R. Bittar R. S. M.. (2020). Magnetic resonance angiography and transcranial Doppler ultrasound findings in patients with a clinical diagnosis of vertebrobasilar insufficiency. Clinics 75:e1212. 10.6061/clinics/2020/e1212 31967281 PMC6963160

[B21] Markus H. S. van der Worp H. B. Rothwell P. M. (2013). Posterior circulation ischaemic stroke and transient ischaemic attack: diagnosis, investigation, and secondary prevention. Lancet Neurol. 12, 989–998. 10.1016/S1474-4422(13)70211-4 24050733

[B22] Mattle H. P. Arnold M. Lindsberg P. J. Schonewille W. J. Schroth G. (2011). Basilar artery occlusion. Lancet Neurol. 10, 1002–14. 10.1016/S1474-4422(11)70229-0 22014435

[B23] Merwick A. Werring D. (2014). Posterior circulation ischaemic stroke. BMJ 348:g3175. 10.1136/bmj.g3175 24842277

[B24] Park J. H. Kim J. M. Roh J. K. (2007). Hypoplastic vertebral artery: frequency and associations with ischaemic stroke territory. *J. Neurol. Neurosurg*. Psychiatry 78, 954–958. 10.1136/jnnp.2006.105767 17098838 PMC2117863

[B25] Park M. G. Choi J. H. Yang T. I. Oh S. J. Baik S. K. Park K. P.. (2014). Spontaneous isolated posterior inferior cerebellar artery dissection: rare but underdiagnosed cause of ischemic stroke. J. Stroke Cerebrovasc. Dis. 23, 1865–1870. 10.1016/j.jstrokecerebrovasdis.2014.02.023 24809669

[B26] Paul N. L. M. Simoni M. Rothwell P. M. (2013). Transient isolated brainstem symptoms preceding posterior circulation stroke: a population-based study. Lancet Neurol. 12, 65–71. 10.1016/S1474-4422(12)70299-5 23206553 PMC3530272

[B27] Pollak L. (2012). Treatment efficacy in vertebrobasilar transient ischemic attacks presenting as isolated vertigo: a retrospective case study. J. Behav. Brain Sci. 2, 92–96. 10.4236/jbbs.2012.21010

[B28] Räty S. Virtanen P. Ritvonen J. Georgiopoulos G. Sairanen T. Lindsberg P. J.. (2024). Thrombolysis in basilar artery occlusion: outcomes and comparison with endovascular thrombectomy. Neurology 102:e209249. 10.1212/WNL.0000000000209249 38531004

[B29] Rothwell P. M. Matthew F. G. Arvind C. Lars M. Olivia G. Jessica N. E. R.. (2007). Effect of urgent treatment of transient ischaemic attack and minor stroke on early recurrent stroke (EXPRESS study): a prospective population-based sequential comparison. Lancet 370, 1432–1442. 10.1016/S0140-6736(07)61448-2 17928046

[B30] Tarnutzer A. A. Berkowitz A. L. Robinson K. A. Hsieh Y. H. Newman-Toker D. E. (2011). Does my dizzy patient have a stroke? A systematic review of bedside diagnosis in acute vestibular syndrome. CMAJ 183, E571–E592. 10.1503/cmaj.100174 21576300 PMC3114934

[B31] Tuna M. A. Rothwell P. M. (2021). Diagnosis of non-consensus transient ischaemic attacks with focal, negative, and non-progressive symptoms: population-based validation by investigation and prognosis. Lancet 397, 902–912. 10.1016/S0140-6736(20)31961-9 33676629 PMC7938377

[B32] Yao K. Zu H. B. (2023). Isolated transient vertigo due to TIA: challenge for diagnosis and therapy. J. Neurol. 270, 769–779. 10.1007/s00415-022-11443-x 36371598

