# Supplementary material for: Vertigo and dizziness due to vertebrobasilar TIA: a prospective study
Source: Front Stroke. 2024 Oct 2;3:1429068. doi: 10.3389/fstro.2024.1429068 (PMC12802699; doi:10.3389/fstro.2024.1429068)
Supplement: Supplementary file 1 [file Data_Sheet_1.PDF]

Patient	Age	Sex	Time from onset (month)	Clinic features	Crises duration (	Triggers	Other hypoflow symptoms	Frequency of episodes (	Comorbidities	Imaging findings	Treatment	Follow up (months)	New crise (month after treatment begining)
1	80	M	24	Dizzyness	<1	Neck position	Trasient visual loss	Daily	HT, DL, CA	LV4 narrowing	Clopidogrel	36	N
2	80	F	1	Dizzyness	<1		Trasient visual loss	1 to 4	HT		Clopidogrel	36	N
3	78	M	1	Unsteadiness	<1		New-onset tinnitus	4 to 8	HT	L cerebellar hemisferic stroke	Clopidogrel	36	N
4	72	M	6	Dizzyness	1 a10		Syncope	1 to 4	HT, DM		Clopidogrel	6	N
5	70	F	14	Dizzyness	>60		Trasient visual loss and syncope	1 to 4	HT	LV1 narrowing	Clopidogrel	16	N
6	60	F	13	Vertigo spining	1 to 10	Neck position	Syncope	4 to 8		LV dissection	Aspirine	36	N
7	55	F	20	Dizzyness	1 to 10	Physical exercise	Left arm paresthesia	1 to 4	DM	L cerebellar hemisferic stroke	Clopidogrel	8	N
8	67	F	<1	Vertigo spining	10 to 60		Cranio-cervical paresthesia	Single	HT, DM, DL	RV4 narrowing	Aspirine	13	N
9	79	M	8	Dizzyness	1 to 10		Trasient visual loss and balance-related fall	<1	HT, IHD		Aspirine	14	N
10	67	F	4	Vertigo spining	1 to 10		Diplopia	4 to 8	HT, DL		Aspirine	10	N
11	70	F	30	Unsteadiness	1 to 10		Trasiente visual loss and dysarthria	<1	HT, DM, DL		Aspirine	12	N
12	85	F	22	Unsteadiness	<1		Trasient visual loss	1 to 4	IHD		Aspirine	20	N
13	69	F	19	Vertigo spining	<1		Trasient visual loss	4 to 8			Aspirine	15	N
14	84	M	16	Dizzyness	<1		Diplopia	Daily	HT, DL		Aspirine	12	N
15	72	F	18	Vertigo spining	<1		Trasient visual loss	1 to 4	HT, IHD		Aspirine	16	N
16	66	F	16	Vertigo spining	1 to 10		Diplopia	1 to 4	HT, DM, DL		Aspirine	16	N
17	61	F	5	Unsteadiness	>60		Diplopia	4 to 8	HT, DM, DL, IHD		Aspirine	22	N
18	65	M	24	Vertigo spining	1 to 10		Trasiente visual loss	<1	HT, DL, stroke	RV4 narrowing	Aspirine	14	N
19	69	F	12	Dizzyness	1 to 10	Neck position	Trasient visual loss and syncope	<1	HT, K-insuf		Aspirine	19	N
20	55	M	12	Vertigo spining	<1		Dysphagia	1 to 4		LV severe hypoplasia	Aspirine	14	N
21	58	M	24	Positional vertigo	1 to 10	Positioning	Trasient visual loss	1 to 4	DL		Aspirine	0	Cerebellar stroke and deth in the day after
22	75	F	1	Dizzyness	<1		Syncope	1 to 4		RV agenesis	Clopidogrel	12	N
23	71	F	1	Vertigo spining	>60			1 to 4	DL, CA		Aspirine	36	N
24	67	F	6	Vertigo spining	<1		Syncope	<1	DM		DAPT	14	N
25	83	M	18	Dizzyness	>60		Syncope	1 to 4	HT, IHD	LV1 narrowing	DAPT	36	N
26	51	M	15	Dizzyness	1 to 10		Trasient visual loss, dysphagia and dysarthria	4 to 8	HT, DM, DL, stroke	R cerebellar sroke, proximal basilar narrowing	DAPT	25	N
27	77	M	60	Vertigo spining	<1	Neck position	Trasient visual loss	Daily	HT, DM, DL, IHD, stroke	R cerebellar stroke, RV severe hypoplasia	DAPT	12	N
28	67	F	18	Dizzyness	1 to 10		Trasient visual loss	4 to 8	HT, DM, DL		DAPT	5	N
29	76	F	2	Dizzyness	>60		Dysphagia	4 to 8	HT, DM, DL, stroke	RV4 narrowing	Aspirine	24	N
30	63	M	24	Vertigo spining	<1		New-oncet cranial-pain	4 to 8	smoking		DAPT	14	N
31	75	M	1	Unsteadiness	>60			Single	HT, DM		DAPT	30	N
32	64	M	4	Vertigo spining	10 to 60		Diplopia	Single	HT, DM, DL		DAPT	6	N
33	69	M	2	Dizzyness	1 to 10		New-oncet cranial-pain	Single	HT	L cerebellar hemisferic stroke	Aspirine	28	N
34	72	M	30	Vertigo spining	1 to 10	Neck position	Transient visual loss	1 to 4	HT, DM		DAPT	24	N
35	83	F	<1	Dizzyness	<1		Syncope	1 to 4	HT, DM		DAPT	25	N
36	71	M	12	Dizzyness	10 to 60		Transient visual loss and syncope	1 to 4	HT, DM	RV severe hypoplasia	Clopidogrel	18	N
37	60	M	6	Vertigo spining	1 to 10	Neck position	Trasient visual loss	4 to 8	HT, DL		Clopidogrel	15	N
38	73	F	6	Vertigo spining	1 to 10	Neck position	Syncope	1 to 4	DM, DL, CA		Clopidogrel	12	N
39	80	F	6	Dizzyness	1 to 10		Hemiparesis	1 to 4	HT, CA		Rivaroxaban	13	N
40	58	M	<1	Dizzyness	10 to 60		Transient visual loss and Dysphagia	1 to 4	HT, DM, DL	L thalamic stroke	DAPT	34	TIA(4)
41	60	F	60	Dizzyness	<1		Transient visual loss and spatial desorientation	<1	HT, DM, DL		DAPT	24	TIA(4)
42	84	M	4	Dizzyness	1 to 10			1 to 4	HT, DL, CA	LV4 narrowing	Clopidogrel	15	N
43	72	F	3	Dizzyness	10 to 60		Trasient visual loss and syncope	<1			Clopidogrel	18	N
44	72	F	5	Vertigo spining	<1		Balance-related fall	1 to 4	HT, DM, DL	R thalamic stroke, RV severe hypoplasia	DAPT	15	N
45	81	F	1	Unsteadiness	10 to 60		Truncal ataxia and syncope	1 to 4	HT, DL	RV2-3 narrowing	DAPT	19	N
46	78	F	5	Dizzyness	<1		Transient visual loss and balance-related fall	1 to 4	HT, DL, CA		Aspirine	17	N
47	79	M	4	Dizzyness	<1	Physical exercise	Syncope	4 to 8	DM, IHD	L cerebellar lacunar stroke, LRV hypoplasia and PC fetal-type	DAPT	15	N
48	66	F	12	Positional vertigo	<1	Positioning	Syncope	1 to 4	HT	RV severe hypoplasia	Asprine	12	N
49	79	M	7	Vertigo spining	1 to 10		Transient visual loss and syncope	1 to 4	HT, DM, IHD		DAPT	6	N
50	80	M	<1	Dizzyness	1 to 10		Syncope	1 to 4	HT, DM	R ant pontine lacunar stoke	DAPT	13	TIA(13)
51	72	F	4	Vertigo spining	>60	Neck position		1 to 4	HT, DL		Aspirine	15	N
52	72	M	8	Dizzyness	1 to 10		Syncope	4 to 8			Aspirine	4	N
53	84	F	2	Dizzyness	>60		Diplopia	Single	HT, DM, CA		DAPT	8	N
54	40	F	<1	Dizzyness	>60		New-oncet cranial-pain and hemiparesis	Single	HT	R pontine stroke, RV dissection	Asprine	24	N
55	73	M	10	Vertigo spining	<1			1 to 4	HT, DM	RV severe hypoplasia	Aspirine	6	N
56	58	M	<1	Vertigo spining	>60		Truncal ataxia	Single			Aspirine	4	N
57	74	F	36	Vertigo spining	<1		Trasient visual loss	1 to 4	HT, DLP	RV1-2 narrowing	Aspirine	13	N
58	72	F	2	Vertigo spining	>60			Single	DM	RV4 narrowing	Aspirine	10	N
59	75	F	3	Vertigo spining	>60		Dysphagia	4 to 8	DM	RV1 narrowing	Clopidogrel	11	N
60	54	M	14	Dizzyness	>60			<1	OSA	R midbrain stroke	Aspirine	6	N
61	79	F	5	Dizzyness	1 to 10	Neck position	Balance-related fall	1 to 4	HT, DL, CA	RV4 narrowing	Aspirine	5	N
62	66	F	60	Vertigo spining	<1		Transient visual loss	1 to 4	HT, DM, DL, smoking		Aspirine	4	N
63	80	M	<1	Dizzyness	10 to 60			Daily			Clopidogrel	6	N
64	75	M	36	Unsteadiness	<1		Balance-related fall	1 to 4	HT, DM, CA		Clopidogrel	10	N
65	77	M	2	Vertigo spining	<1		Balance-related fall	4 to 8	HT, DL	R ant pontine lacunar stoke	Aspirine	7	N
66	67	M	<1	Vertigo spining	>60		Diplopia	Single	HT, DM	L cerebellar stroke	Aspirine	5	N
67	70	F	<1	Vertigo spining	>60		Syncope	Single	HT		Clopidogrel	13	N
68	78	F	2	Unsteadiness	1 to 10		Dysarthria	Single	DL, smoking		Aspirine	9	N
69	57	F	12	Dizzyness	10 to 60	Physical exercise	Transient visual loss, syncope	<1	HT, DM	L Medulla-pons region stroke	Aspirine	8	TIA(2)
70	75	M	18	Vertigo spining	1 to 10		Syncope	Daily	HT, IHD, stroke		Aspirine	4	N
71	61	F	5	Dizzyness	1 to 10	Neck position	Transient visual loss	4 to 8	HT, DL, stroke		DAPT	6	N
72	70	M	36	Vertigo spining	>60		Syncope	<1	HT, OSA	Bilateral V hypoplasia and bilateral PCa fetal-type	Aspirine	7	N
73	82	F	7	Dizzyness	1 to 10			4 to 8	DM, DL, CA, K-insuf		DAPT	6	TIA(4)
74	60	F	2	Dizzyness	<1		Dysphagia and new-oncet cranial-pain	1 to 4	DM	R thallamic stroke, RV4 narrowing	Aspirine	3	N

75	72	M	24	Dizzyness	1 to 10		Diplopia and facial paresthesis	1 to 4	HT	R cerebellar lacunar stroke	Clopidogrel	8	N
76	80	M	9	Vertigo spining	1 to 10			1 to 4	HT, DL, OSA		DAPT	4	N
77	79	F	24	Dizzyness	<1	Neck position and physical exercise	Balance-related fall	1 to 4	HT, DL, stroke	LV1 narrowing	DAPT	7	N
78	73	F	2	Dizzyness	<1	Neck position	Syncope	1 to 4	HT, DL, K-insuf	L cerebellar stroke, RV severe hyoplasia	Aspirine	5	N
79	84	F	<1	Dizzyness	1 to 10		Transient visual loss	4 to 8	HT, DL	LV severe hypoplasia	Clopidogrel	12	N
80	73	M	6	Vertigo spining	1 to 10	Physical exercise	Transient visual loss and syncope	Daily	HT, DM, DL, smoking		DAPT	6	N
81	84	M	<1	Dizzyness	1 to 10		Transient visual loss	4 to 8	HT, DM, DL, IHD	LV severe hypoplasia, RV4 narrowing	Rivaroxaban	4	N
82	81	F	1	Unsteadiness	10 to 60		Diplopia	Single	DL		Aspirine	11	N
83	76	M	2	Vertigo spining	10 to 60		Syncope	1 to 4	HT, DM, DL	LV severe hypoplasia	Aspirine	12	N
84	74	F	18	Unsteadiness	<1			4 to 8	HT		Aspirine	3	N
85	75	M	36	Unsteadiness	>60		Balance-related fall and spatial desorientation	<1	HT		Aspirine	5	N
86	71	M	<1	Dizzyness	10 to 60		Diplopia	Single	DM, DL, OSA	LV1,4 narrawing, RV1 narrowing	Aspirine	4	N
87	74	M	3	Vertigo spining	10 to 60		Truncal ataxia, spatial desorientation	4 to 8	HT, DM		Aspirine	13	N
88	37	F	<1	Dizzyness	10 to 60		Limb ataxia and facial paresthesis	Single		LV4 narrowing	Aspirine	5	N
89	82	F	36	Dizzyness	10 to 60		Syncope	<1	Ht, DL	RV4 narrowing	Aspirine	4	N
90	56	F	36	Dizzyness	<1		Syncope	1 to 4	HT, DM, DL	RV1 narrowing	Aspirine	12	N
91	57	F	<1	Vertigo spining	10 to 60		Transient visual loss and syncope	Single	DM		Aspirine	13	N
92	79	F	2	Dizzyness	1 to 10			1 to 4	HT, DM, DL, smoking	Bilateral vertebral dolichoectasia	Aspirine	7	N
93	71	F	8	Dizzyness	<1		Balance-related fall	<1	HT, DL	Bilateral V hypoplasia and bilateral PCa fetal-type	Clopidogrel	12	N
94	70	F	<1	Positional vertigo	<1	Positioning	Diplopia and new-oncet cranial-pain	Single	HT, DM, stoke		DAPT	2	Cerebellar stroke in the day after
95	80	F	12	Dizzyness	<1		Balance-related fall	1 to 4	HT		Aspirine	3	N
96	70	F	72	Vertigo spining	<1	Neck position		4 to 8	HT, DM, DL		Aspirine	4	N
97	59	F	3	Vertigo spining	>60		Hemiparesis	<1	HT, IHD, stroke	L cerebellar hemisferic stroke	DAPT	4	N
98	60	M	2	Dizzyness	>60			<1			Aspirine	6	N
99	75	M	20	Vertigo spining	1 to 10		Transient visual loss	1 to 4	HT, DM, OSA		Aspirine	3	N
100	80	M	12	Dizzyness	<1			1 to 4	HT, DM, DL		Aspirine	3	N
101	74	M	6	Vertigo spining	1 to 10		Limb ataxia	1 to 4	HT, DL		Aspirine	3	N
102	73	F	3	Vertigo spining	<1			1 to 4	DM, CA		Aspirine	4	N
103	69	F	<1	Vertigo spining	<1		Syncope	Single	HT, DL		Aspirine	7	N

DL - dyslipidemia
DM - diabetes
IHD - ischemic heart disease
CA - cardiac arrhythmia
OSA - obstructive sleep apnea
K-insuf - kidney insufficience
L - left side
R - right side
V - vertebral artery
V4 - 4th vertebral artery's segment
V1 - 1st vertebral artery's segment
PCa - posterior cerebral artery
V2 - 2nd vertebral artery's segment
V3 - 3rd vertebral artery's segment
DAPT - double antiplatelet therapy
TIA - transient ischemic attack
