# Supplementary material for: Vertigo and dizziness due to vertebrobasilar TIA: a prospective study
Source: Front Stroke. 2024 Oct 2;3:1429068. doi: 10.3389/fstro.2024.1429068 (PMC12802699; doi:10.3389/fstro.2024.1429068)
Supplement: Supplementary file 2 [file Data_Sheet_2.PDF]

Fig. S2. Vertebrobasilar circulation.

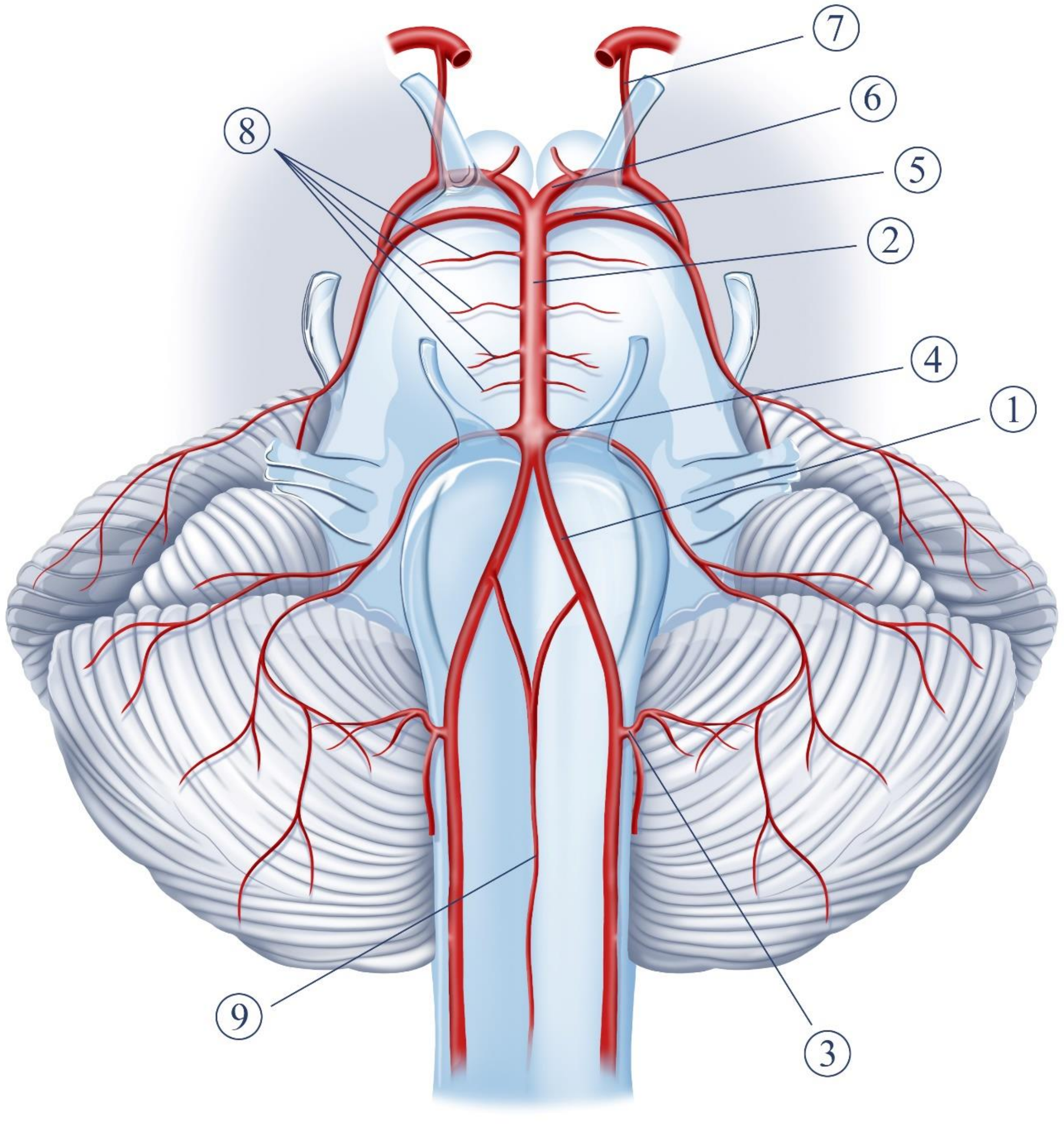

1 – vertebral artery, 2 – basilar artery, 3 – posterior inferior cerebellar artery, 4 – anterior inferior cerebellar artery, 5 – superior cerebellar artery, 6 – posterior cerebral artery, 7 – posterior communicating artery, 8 – pontine arteries, 9 – medullary artery.
